# Can we characterize A-P/IAP behavioural phenotypes in people with chronic pain?

**DOI:** 10.3389/fpain.2023.1057659

**Published:** 2023-02-15

**Authors:** Vaidhehi Veena Sanmugananthan, Joshua C. Cheng, Kasey S. Hemington, Anton Rogachov, Natalie Rae Osborne, Rachael L. Bosma, Junseok Andrew Kim, Robert D. Inman, Karen Deborah Davis

**Affiliations:** ^1^ Division of Brain, Imaging and Behaviour, Krembil Brain Institute, Krembil Research Institute, University Health Network, Toronto, ON, Canada; ^2^ Institute of Medical Science, University of Toronto, Toronto, ON, Canada; ^3^ Department of Medicine, University of Toronto, Toronto, ON, Canada; ^4^ Department of Surgery, University of Toronto, Toronto, ON, Canada

**Keywords:** behavioural phenotype, chronic pain, attention, reaction time, rumination

## Abstract

Two behavioural phenotypes in healthy people have been delineated based on their intrinsic attention to pain (IAP) and whether their reaction times (RT) during a cognitively-demanding task are slower (*P*-type) or faster (A-type) during experimental pain. These behavioural phenotypes were not previously studied in chronic pain populations to avoid using experimental pain in a chronic pain context. Since pain rumination (PR) may serve as a supplement to IAP without needing noxious stimuli, we attempted to delineate A-P/IAP behavioural phenotypes in people with chronic pain and determined if PR can supplement IAP. Behavioural data acquired in 43 healthy controls (HCs) and 43 age-/sex-matched people with chronic pain associated with ankylosing spondylitis (AS) was retrospectively analyzed. A-P behavioural phenotypes were based on RT differences between pain and no-pain trials of a numeric interference task. IAP was quantified based on scores representing reported attention towards or mind-wandering away from experimental pain. PR was quantified using the pain catastrophizing scale, rumination subscale. The variability in RT was higher during no-pain trials in the AS group than HCs but was not significantly different in pain trials. There were no group differences in task RTs in no-pain and pain trials, IAP or PR scores. IAP and PR scores were marginally significantly positively correlated in the AS group. RT differences and variability were not significantly correlated with IAP or PR scores. Thus, we propose that experimental pain in the A-P/IAP protocols can confound testing in chronic pain populations, but that PR could be a supplement to IAP to quantify attention to pain.

## 1. Introduction

Pain is an attention-grabbing sensory experience, and acute pain plays a role in protecting our bodies from harm (1, 2). Pain and attention are interconnected. For example, allocation of attention can modulate components of the pain experience (e.g., pain intensity) and activation of pain-related brain regions (3–11). Much of the research that has examined pain and attention interactions has been conducted in healthy individuals, but studies in chronic pain populations are challenging and confounded by the need to apply an experimental pain stimulus in the context of chronic pain.

Our lab has demonstrated that healthy individuals vary in their ability to balance attention towards pain vs. other attentional demands, and that these differences generally can be observed by two behavioural phenotypes. One behavioural phenotype is based on how performance of an attention-demanding task is impacted by concurrent experimental acute pain stimuli. We have designated individuals as either “Attention to task dominants” (A-types) for those that exhibit faster task reaction times (RTs) or “pain dominants” (P-types) for those that exhibit slower task RTs during concurrent pain stimulation compared to a no-pain condition (10, 12–14). The other behavioural phenotype is based on an assessment we developed to determine intrinsic attention to pain (IAP), which reflects the tendency of an individual to attend to an acute pain stimulus vs. mindwander away from the pain stimulus (1, 15). Healthy individuals designated into these two behavioural phenotypes have been shown to have characteristic structural and functional attributes in areas of the dynamic pain connectome in the brain (1, 10, 12–15).

Behavioural phenotypes that identify how an individual attends to pain could provide some individual predictive value of the potential effectiveness of attentional or psychotherapeutic interventions that aim to alter attentional engagement towards chronic pain (e.g., cognitive behavioural therapy) (16, 17). However, A-P types and IAP have not been characterized in chronic pain populations in part because of potential confounds associated with applying experimental pain stimuli in a chronic pain context and the unknown relevancy of using experimental pain as a model for chronic pain (18–20).

In this exploratory study, our main aim was to examine A-P and IAP behavioral phenotypes in people with chronic pain using our standard A-P/IAP protocols that use acute experimental pain. However, because of the inherent confound of applying experimental pain in a chronic pain context, our secondary exploratory aim was to explore whether pain rumination (PR) could be used to quantify attention to pain and provide an experimental pain-free supplement to IAP.

PR is repetitive and continuous negative thinking about pain and the possible causes and consequences associated with it's experience (21). PR is thought to be related to IAP (1, 21) because it also captures an individual's tendency to attend to pain. However, the relationship between these two metrics has not been examined previously.

## 2. Materials and methods

### 2.1. Participants

This study comprised a retrospective analysis of behavioural data collected from 43 right—handed people with chronic pain associated with ankylosing spondylitis (AS) (average age = 28.7, SD = +/−6.4 years old; 30 males, 13 females) and 43 age- and sex- matched pain-free healthy controls (HC) (average age = 28.2, SD = +/−6.1 years old; 30 males, 13 females) with the overall ages ranged from 18 to 40 (+/−2) years old.

All study participants provide informed consent to experimental methods that were approved by the University Health Network Research Ethics Board. We recruited individuals with AS from the Toronto Western Hospital's Spondylitis Clinic and that were diagnosed with AS using the modified New York criteria (22, 23). Both HCs and AS participants were excluded if they met any of the following conditions: (1) current or were previously diagnosed with a psychiatric, neurological, or metabolic disorder, (2) previous major surgeries, (3) any serious infection within 4 weeks of data collection requiring hospitalization and/or antibiotics.

### 2.2. Tasks

#### 2.2.1. Numeric interference task

Participants were familiarized with a numeric interference (NI) task (see 12, 13, 24–27) and underwent a training session before testing began. The NI task required the participants to view a computer screen that displayed 3 separate boxes, each of which contained a different number of digits that ranged in value from 1 to 9. Within each box there were identical numbers but there were different numbers across the boxes. Each participant was instructed to use a numerical keyboard to indicate as quickly and as accurately as possible, the highest number of digits across the boxes. The cognitive-demanding aspect of the task was that participants had to report the highest number of digits (non-dominant information) rather than the highest number value (dominant information) (12, 13, 24–27). The study included 6 blocks with 24 trials each (trial length = 2.5 s, inter-block interval =60 s), and blocks alternated between a no-pain condition and a pain condition during which experimental pain was applied concurrently during the task (12, 13). A computer-controlled transcutaneous electrical nerve stimulation (TENS) device (300-PV Empi Inc.) was used to deliver stimuli to the left median nerve and was calibrated prior to testing to elicit pain intensity of approximately 40–60/100 (0 = no pain, 100 = most intense pain imaginable) for each participant. The NI task was run on EPrime v1.1 (Psychological Software tools). See the Supplementary Materials for more details about the TENS stimulus calibration procedure. The first two blocks of the NI task (one no-pain block and one pain block) were removed to avoid learning effects for each participant.

#### 2.2.2. Measuring performance on the NI task and data-cleaning

Task performance was quantified from each participants' mean RT and RT variability (RTv) (12) across the no-pain and pain blocks, respectively (see our previous study 12). The RTv in each participant was calculated from the variance of the RTs in all of the trials of the no-pain blocks and the pain blocks separately.

Trials with RTs that were <=200 milliseconds (ms) or >=2500 ms were removed. The upper cut-off was determined based on the maximum trial time. The lower cut- off was based on the postulated time needed for physiological processes (e.g., stimulus detection, decision making, motor response) to occur (approximately 100–200 ms) during a reaction-time (28, 29). Participant exclusion criteria was set at having more than 30% of their total trials missing from each block-type and/or all blocks together after data-cleaning was completed. No participants were excluded from analyses after the data-cleaning procedure was implemented.

#### 2.2.3. A-P categorization of individuals

The differences in RT between the no-pain blocks and the pain blocks of the NI task were used to characterize A and P types as we have done in our previous studies: The RTmean of the no-pain blocks was subtracted from the RTmean of the pain blocks (ΔRTmean = RTmean pain—RTmean no pain) for each participant separately (12, 13). Thus, the A-types exhibit negative ΔRTmean values which reflect a general increase in task performance speed from the no-pain to pain condition of the NI task, whereas the P-types exhibit positive ΔRTmean values, which reflect a general decrease in task performance speed from the no-pain to pain condition of the NI task.

### 2.3. Quantifying attention to pain

We used two approaches to quantify attention to pain; the IAP measure we have developed in our lab that uses an experimental stimulus, and an assessment of pain rumination which is a measure that does not require applying stimuli:

Participants underwent an experience sampling of experimental pain stimuli previously developed by Kucyi et al. (15) to quantify an individual's IAP. To do this, participants were asked to stare at a blank screen with a white fixation cross during which a 20 s transcutaneous electrical stimulus was delivered to the skin overlying the left median nerve (300-PV, Empi Inc.) at an intensity to evoked pain rated at 40–60/100 (0 = no pain, 100 = most intense pain imaginable) that was calibrated prior to the task for each participant separately. See the Supplementary Materials for more information regarding the stimulus calibration procedure. After 20 s, the pain stimulus stopped, and a probe popped up on the screen that asked participants to indicate whether their attention had been “only on pain”, “mostly on pain”, “mostly on something else”, or “only on something else”. After the participants responded to this prompt or after 8 s had passed, an inter-stimulus interval with the blank screen and white fixation cross popped up without pain for 22 s. In total, participants underwent 20 trials of this task. Based on the proportions of trials that reported attention towards pain vs. attention towards something else, a single IAP score was calculated for each participant that ranged from −2 (always attending to something else) to +2 (always attending to pain) as follows (15):

$$\begin{array}{rl} \mathrm{IAP}= & \;[(2n_{only\;pain}+n_{mostly\;pain})\;\text{–}\;(2n_{only\;else}+n_{mostly\;else})]/(n_{total}) \end{array}$$

where *n* = number of trials

We quantified PR using the 4 item pain rumination subscale of the pain catastrophizing scale (PCS-R). The entire PCS consists of 13 items, each of which is rated on a five-point Likert Scale (0—not at all, 4- all the time). A score between 0 (lowest PR score) and 16 (highest PR score) was generated for each participant based on their responses.

### 2.4. Analyses

Analyses were conducted using R-Studio, Graphpad-Prism 7, and Microsoft Excel. We used parametric and non-parametric tests as appropriate: Independent sample t-tests were used to examine differences in RTmean and RTv, respectively, between the HCs and AS group. This was done for each NI task condition (no-pain and pain), separately. The difference in IAP scores between HCs and the AS group were examined using an independent sample t-test. The difference in PCS-R scores between the HCs and the AS group were examined using a Mann-Whitney-u test.

We used Spearman's correlations to determine the correlation between IAP and PCS-R scores in the HCs and the AS group, and for the correlations between (i) ΔRTmean values and IAP scores, (ii) ΔRTmean values and PCS-R scores, (iii) ΔRTv values and IAP scores, and (iv) ΔRTv values and PCS-R scores for the HCs and the AS group.

## 3. Results

### 3.1. Attention to task-dominant (A-type) and pain-dominant (P-type) characterization and performance on the NI task

**Figure 1:**
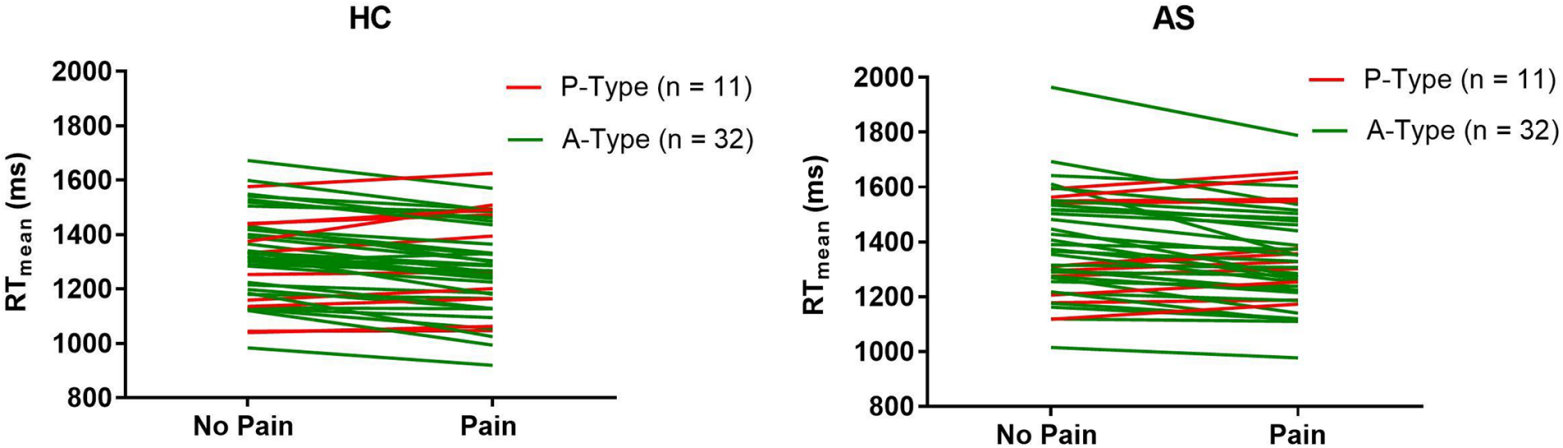
Change in individual mean task reaction times (RT) from the no-pain to the pain blocks for each individual in the healthy controls (HCs) and ankylosing spondylitis (AS) groups. A-types are represented by the green lines. *P*-types are represented by the red lines.

**Figure 2:**
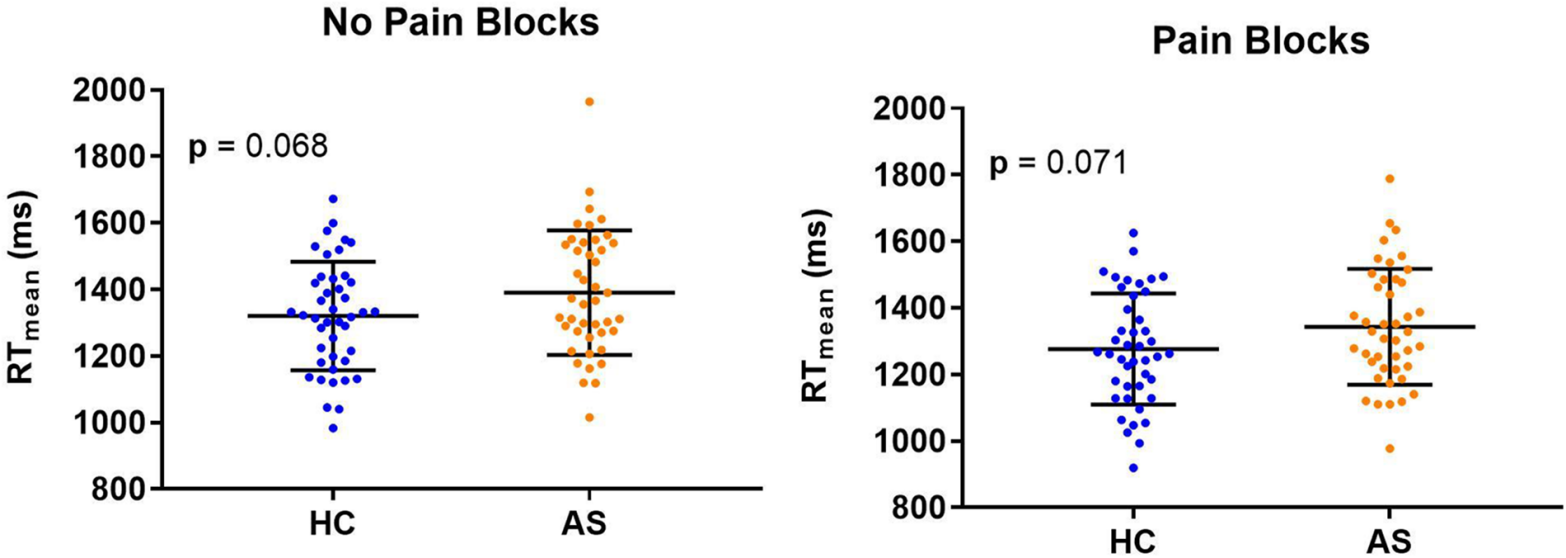
Individual and mean group task reaction times (RTs) for the healthy controls (HCs) and the ankylosing spondylitis (AS) group. Individual HCs are represented by the blue dots and individuals in the AS group are represented by the orange dots.

**Figure 3:**
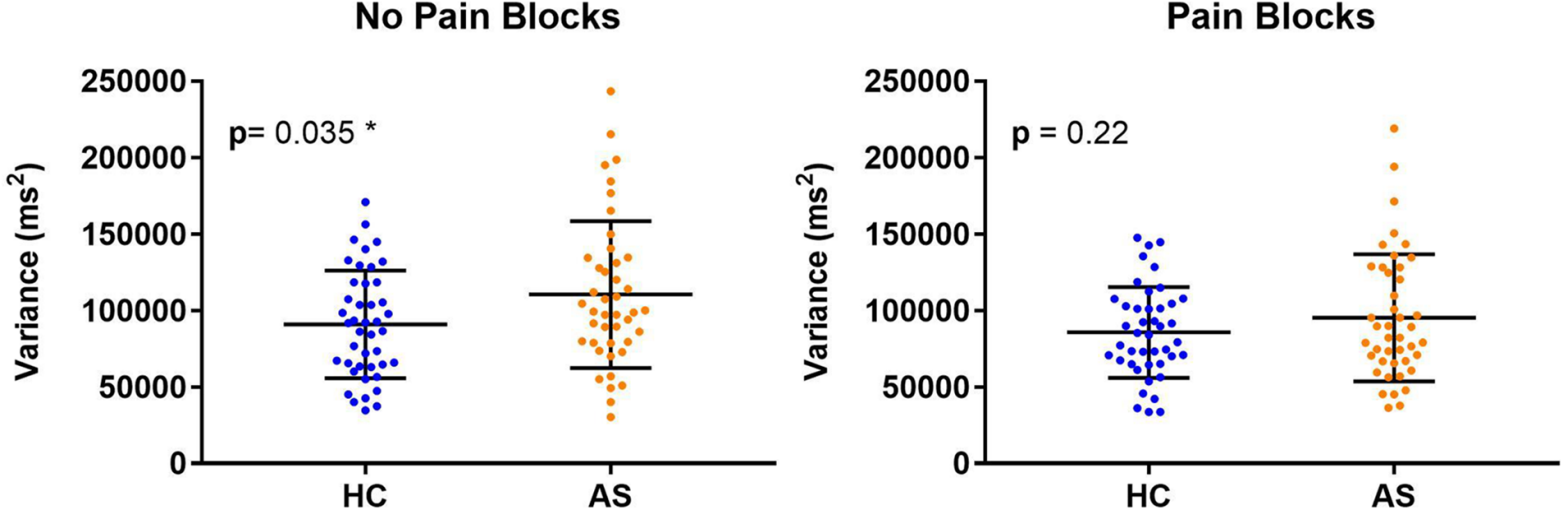
Individual and mean group task reaction time variance (RTv). Higher task RTv was found in the healthy controls (HCs) compared to the ankylosing spondylitis (AS) group in the no-pain blocks (left panel) but not in the pain blocks (right panel). The HCs are represented by the blue dots. The AS group is represented by the orange dots.

Within each cohort of HCs and AS, we delineated 32 A-type individuals and 11 P-type individuals (Figure 1). There were no significant differences in RT mean between the HCs and AS group in either the no-pain condition (HC: M = 1320.47 ms, SD = 163.39 ms; AS: M = 1390.40 ms, SD = 186.64 ms) (*t* = 1.85, *p* = 0.068, Cohen's d = 0.40) or in the pain condition (HC: M = 1276.51 ms, SD = 167.39 ms; AS: M = 1343.93 ms, SD = 173.96 ms) (*t* = 1.83, *p* = 0.071, Cohen's d = 0.39) of the NI task (Figure 2). Furthermore, there was no significant difference in RTv between the HCs and the AS group in the pain condition of the NI Task (HC: M = 8.6 × 10^4^ ms^2^, SD = 3.0 × 10^4^ ms^2^; AS: M = 9.5 × 10^4^ ms^2^, SD = 4.2 × 10^4^ ms^2^) (*t* = 1.24, *p* = 0.22, Cohen's d = 0.27). However, as shown in Figure 3, there was a significant difference in task RTv between the HCs and the AS group in the no-pain condition (*t* = 2.15, *p* = 0.035, Cohen's d = 0.46) such that the AS group exhibited an overall higher mean RTv than the HCs (HC: M = 9.1 × 10^4^ ms^2^, SD = 3.5 × 10^4^ ms^2^; AS: M = 1.1 × 10^5^ ms^2^, SD = 4.8 × 10^4^ ms^2^).

### 3.2. Attention to pain: intrinsic attention to pain and pain rumination

**Figure 4:**
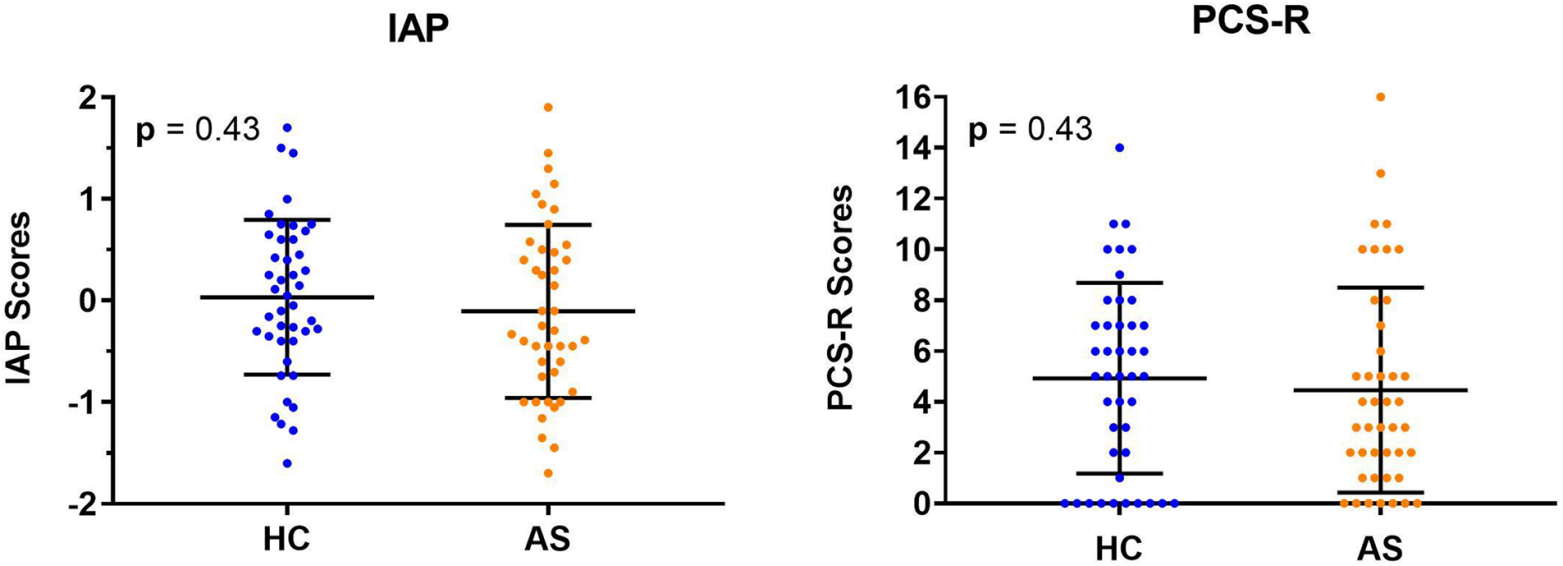
Individual and group mean intrinsic attention to pain (IAP) and pain rumination scores. Neither the IAP scores (left panel) or rumination scores (right panel) from the pain catastrophizing scale, rumination subscale (PCS-R) were significantly different between healthy controls (HCs) and those in the ankylosing spondylitis (AS) group. The HCs are represented by the blue dots. The AS group is represented by the orange dots.

**Figure 5:**
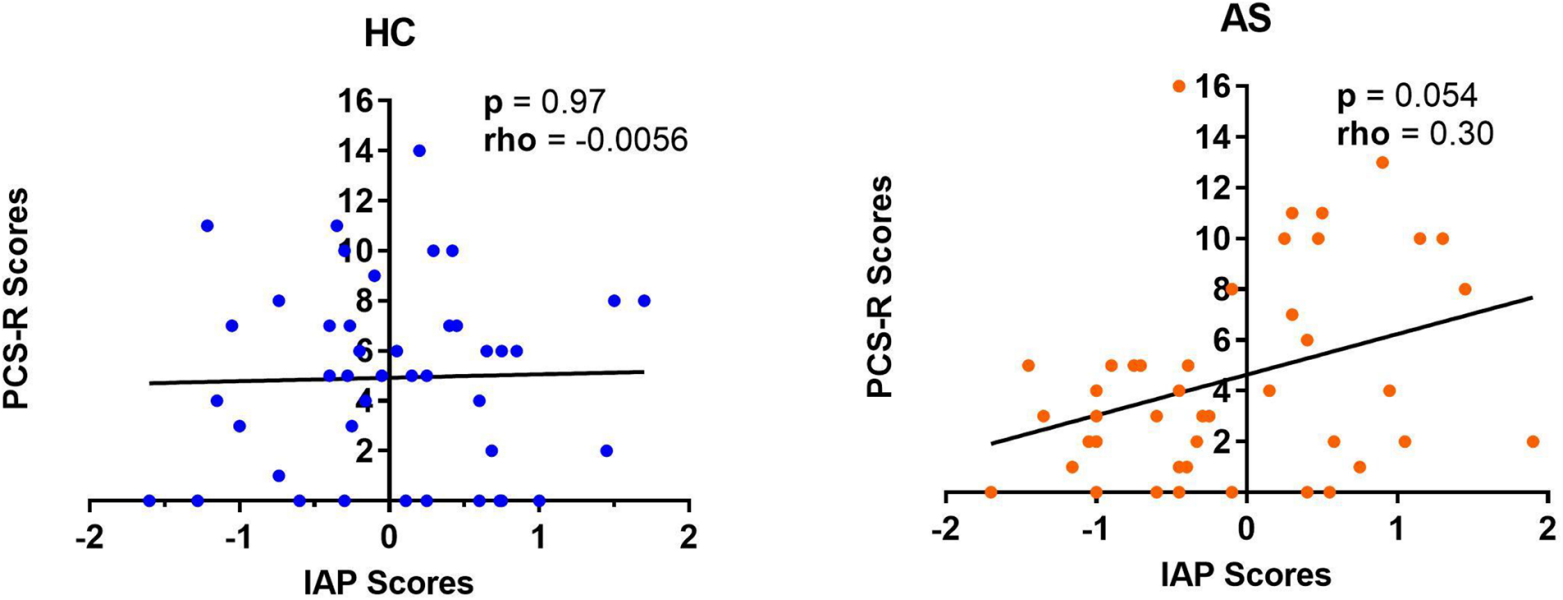
Relationship between intrinsic attention to pain (IAP) and pain rumination. The IAP scores were not significantly correlated with the pain rumination scores (derived from the rumination subscale of the pain catastrophizing scale) (PCS-R) in healthy controls (HCs) (left panel) but there was a marginally significant positive correlation between the IAP scores and the PCS-R scores in the ankylosing spondylitis (AS) group (right panel). The HCs are represented by the blue dots. The AS group is represented by the orange dots.

We examined two metrics of attention to pain: IAP and PCS-R. We did not find any significant group differences in IAP scores (HC: M = 0.033, SD = 0.76; AS: M = −0.11, SD = 0.85) (*t* = 0.80, *p* = 0.43, Cohen's d = 0.17) or in the PCS-R scores (HC: M = 4.93, SD = 3.75; AS: M = 4.47, SD = 4.04) (*p* = 0.43, Cohen's d = 0.12) between the HCs and the AS group (Figure 4). Furthermore, the IAP scores were not significantly correlated with the PCS-R scores in the HCs (rho = −0.0056, *p* = 0.97). However, the correlation between IAP scores and the PCS-R scores in the AS group showed a statistically significant trend (rho = 0.30, *p* = 0.054) (Figure 5).

### 3.3. Relationship between task performance and the metrics of attention to pain

**Figure 6:**
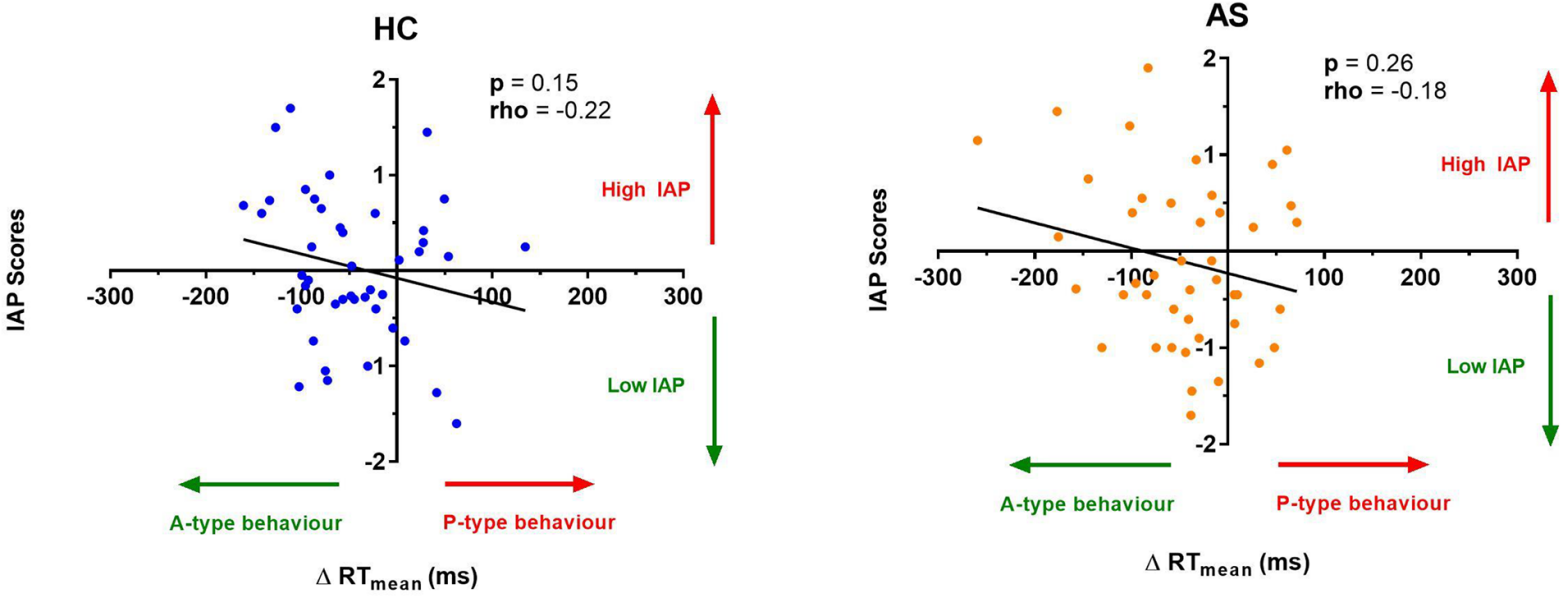
Relationship between an individual's intrinsic attention to pain (IAP) and the effect of pain on task reaction time (RT). No significant correlation was found between the IAP scores and the ΔRT mean in the healthy controls (HCs) (left panel) or for the ankylosing spondylitis (AS) group (right panel). The HCs are represented by the blue dots. The AS group is represented by the orange dots. A-type behavior is represented by a negative ΔRT mean value (values that are on the left side of the horizontal axis). *P*-type behavior is represented by a positive ΔRT mean value (values that are on the right side of the horizontal axis).

**Figure 7:**
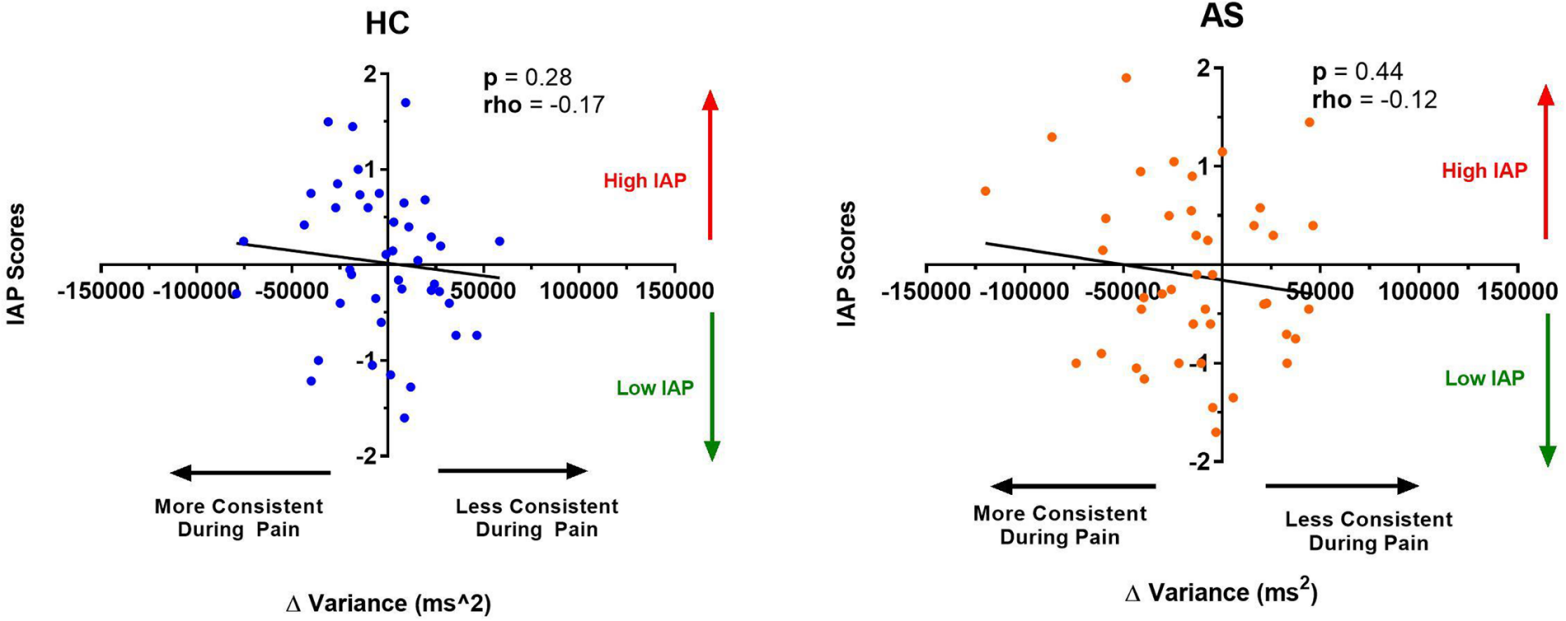
Relationship between an individual's intrinsic attention to pain (IAP) and the effect of pain on variance in task reaction time (RTv). No significant correlation was found between the IAP scores and the ΔRTv in the healthy controls (HCs) (left panel) or for the ankylosing spondylitis (AS) group (right panel). The HCs are represented by the blue dots. The AS group is represented by the orange dots. More consistent RTs during pain is represented by a negative ΔRTv value (values that are on the left side of the horizontal axis). Less consistent RTs during pain is represented by a positive ΔRTv value (values that are on the right side of the horizontal axis).

**Figure 8:**
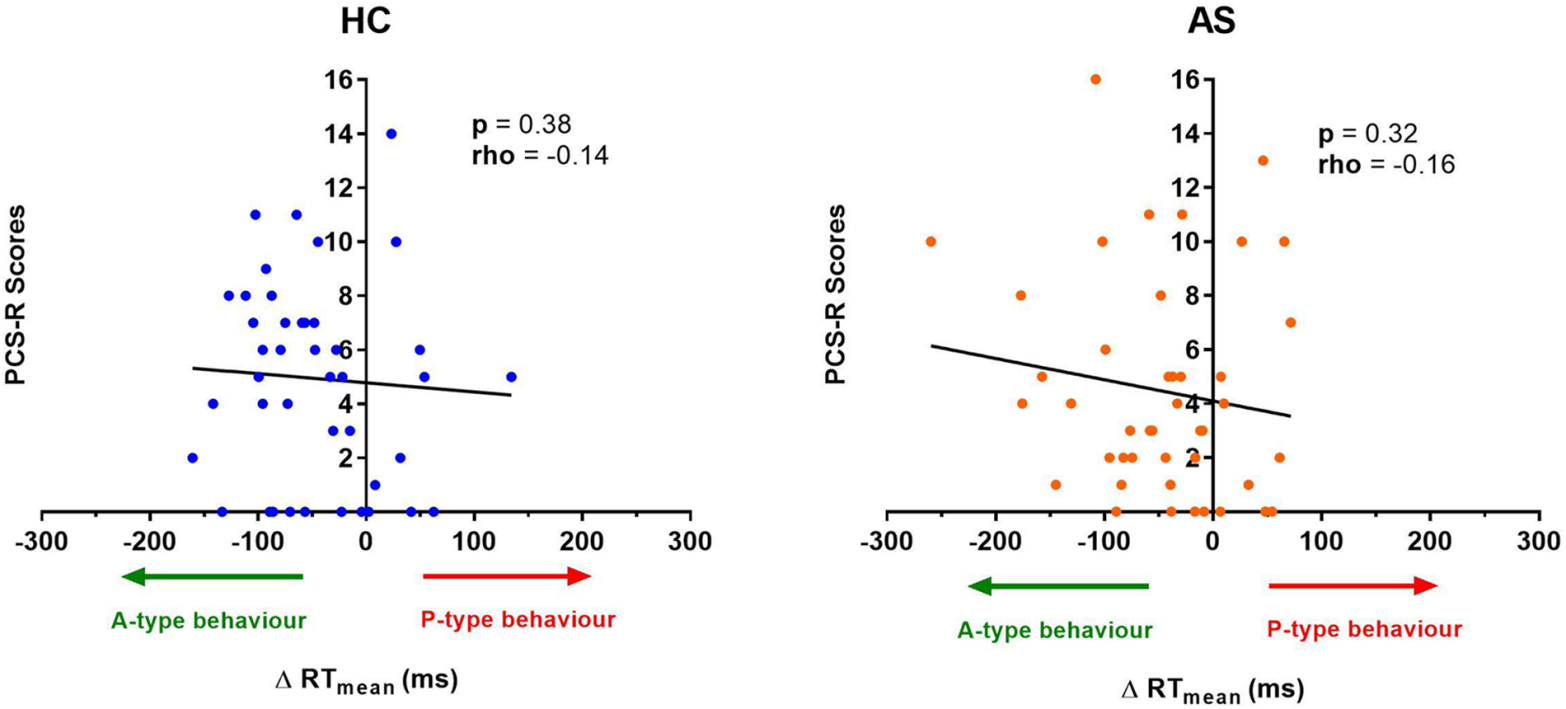
Relationship between an individual's pain rumination score and the effect of pain on their task reaction time (RT). No significant correlation was found between the pain rumination scores (derived from the rumination subscale of the pain catastrophizing scale) (PCS-R) and the ΔRT mean in the healthy controls (HCs) or for the ankylosing spondylitis (AS) group. The HCs are represented by the blue dots. The AS group is represented by the orange dots. A-type behavior is represented by a negative ΔRT mean value (values that are on the left side of the horizontal axis). P-type behavior is represented by a positive ΔRT mean value (values that are on the right side of the horizontal axis).

**Figure 9:**
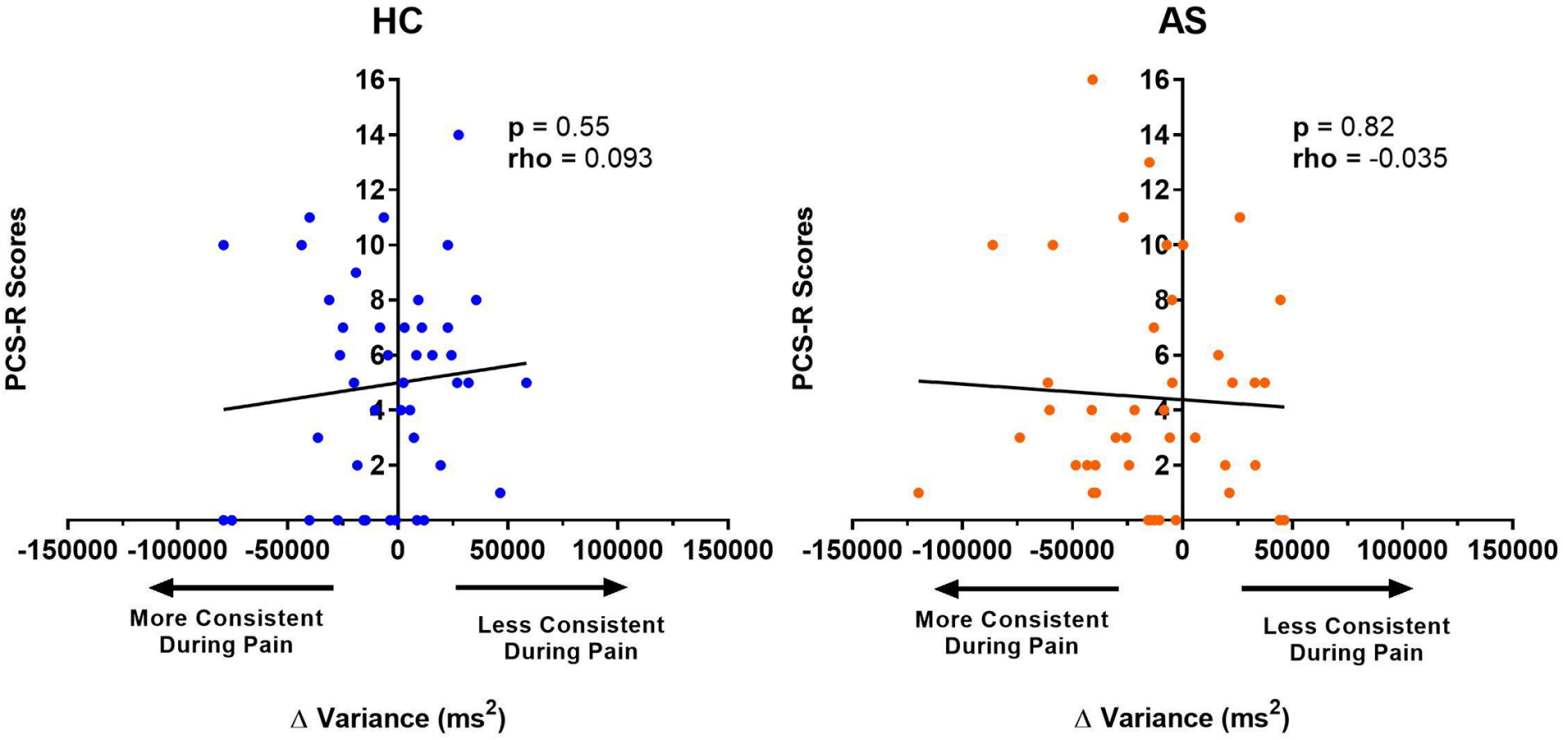
Relationship between an individual's pain rumination score and the effect of pain on their variance in task reaction time (RTv). No significant correlation was found between the pain rumination scores (derived from the rumination subscale of the pain catastrophizing scale) (PCS-R) and the ΔRTv in the healthy controls (HCs) or for the ankylosing spondylitis (AS) group. The HCs are represented by the blue dots. The AS group is represented by the orange dots. More consistent RTs during pain is represented by a negative ΔRTv value (values that are on the left side of the horizontal axis). Less consistent RTs during pain is represented by a positive ΔRTv value (values that are on the right side of the horizontal axis).

We next examine the relationship between task performance and metrics of attention to pain at the individual and group level. We did not find any significant correlation between the IAP scores and ΔRTmean in the HCs (rho = −0.22, *p* = 0.15) or in the AS group (rho = −0.18, *p* = 0.26) (Figure 6). We also examined the consistency of task performance (Figure 7) and again found there was no significant correlation between the IAP scores and ΔRTv in the HCs (rho = −0.17, *p* = 0.28) and the AS group (rho = −0.12, *p* = 0.44). Also, there was no significant correlation between the PCS-R scores and ΔRTmean (Figure 8) in the HCs (rho = −0.14, *p* = 0.38) and the AS group (rho = −0.16, *p* = 0.32). Finally, we also did not find any significant correlations between the PCS-R scores and ΔRTv (Figure 9) in the HCs (rho = 0.093, *p* = 0.55) or in the AS group (rho = −0.035, *p* = 0.82).

### 3.4. Supplemental analysis: pain scores in ankylosing spondylitis group, sex differences in task performance and metrics of attention to pain

As a supplementary exploratory analysis, pain intensity scores at the time of testing and on average over 4 weeks were obtained from the AS group using the painDETECT questionnaire, and compared across the A-and P-types (30, 31). There was no statistically significant differences in current (i.e., state) pain scores across A- and P-types. However, there was a marginally significant difference in average (i.e., trait) pain scores over 4 weeks between the A- and P-types such that P-types had higher overall average pain scores than the A-types (see Supplemental Materials). As well, we examined sex differences in RTmean, RTv, IAP scores and PCS-R scores in the HCs and AS group. There were no statistically significant sex differences identified other than a marginally significant difference in RTv between males and females in the AS group for the pain condition of the NI task (see Supplemental Materials). These findings were not included as part of our main analyses due to low and unequal group sizes between the A-P types and the sexes (arising from the male-predominance of AS).

## 4. Discussion

Categorizing people with the A-P and IAP behavioural phenotypes (10, 12–15) provides insight to understand acute pain and attention interactions but in chronic pain populations this assessment could be confounded by ongoing and fluctuating chronic pain (32–34). Additionally, mechanisms underlying pain-attention interactions may differ for acute and chronic pains. As a first step towards determining the suitability of A-P and IAP testing in chronic pain populations, we characterized A-P and IAP behavioural phenotypes in people with chronic pain associated with AS. Our main findings were that (1) the current A-P and IAP protocols are not suitable for people with chronic pain, and (2) PR can be used as a supplement to IAP to capture attention to pain in chronic pain populations without the need for experimental pain stimuli.

We identified both A and P-type individuals in both the AS group and healthy controls, and there were more A-types than P-types in both groups. This was a surprising revelation, considering that many studies suggest people with chronic pain can demonstrate difficulty attending away from their chronic pain (35–37) and overall impairment in many cognitive domains and tasks (24, 36, 38–40). Therefore, we expected that the AS group's behavioural performance would be affected by other pain experiences like the NI task's concurrent experimental acute pain, and that they would exhibit slower RTs in the task pain condition compared to the no-pain condition. This could have been a result of sampling bias, as A-types might be more likely to volunteer for a pain study than P-types. Another explanation could be that the AS group has built resilience to pain experiences during the course of their disease, thus allowing them to perform better during the NI Task. The AS group may have learned overtime to function normally and accept their pain having been exposed to chronic pain from having AS. Many people with chronic pain build resilience towards their pain, that allows them to operate in their daily lives (41–43). As well, those who have higher acceptance of their chronic pain report lower levels of disability and higher levels of functionality in daily activities than those with lower acceptance (44, 45). The level of resilience and acceptance of chronic pain in the AS group may have supported their ability to perform on the NI task, however we have not tested if this is true.

Similarly to Cheng et al. (12), we used RT variability as a metric of performance to gain insight into inconsistencies in RTs (46), as this measure is considerably understudied in studies looking at pain interference and performance variability (12). Interestingly, there were no differences in RT variances between the HCs and the AS group in the pain condition of the NI task, but there were significant differences in the no-pain condition such that the chronic pain group exhibited overall higher variance compared to the HCs despite there being no experimental pain applied. Since experimental pain is not driving this variance in the AS group, it is possible that the chronic pain experienced by this population could be interfering with the consistency of their RTs on this task. However, our current protocols do not account for fluctuating and spontaneous chronic pain that the AS group may be experiencing during the NI task. To properly determine if an individual is an A or P type, RTs during the task need to be compared in a condition that has no-pain to a condition where pain is concurrently occurring within the behavioural task. Since we cannot verify the occurrence of chronic pain during the NI task, this suggests that these protocols are not appropriate for people with chronic pain and that further modifications need to be made that take chronic pain into consideration.

It is possible the AS group could have experienced an inhibitory pain modulation or “pain inhibits pain” (47) phenomena during the pain condition of the NI task, such that the experimental acute pain inhibited their chronic pain, which allowed them to produce more consistent RTs in the task pain condition compared to the no-pain condition. Including recordings of chronic pain intensity in future iterations of the A-P/IAP protocols would allow us to further explore whether experimental pain inhibits chronic pain experiences during these behavioral tasks.

The marginally significant correlation between IAP scores and PCS-R scores in the AS group suggests PCS-R may be able to quantify “attention to pain” in people with chronic pain, alongside IAP. However, these measures likely quantify different aspects of “attention to pain”. IAP has been shown to be a “trait-like” measure of attention to pain (1, 15), and probes participants to think only about whether their attention was directed towards the administered pain or mind-wandering towards something else. PR is defined as “…perseverative negative thinking about pain” (21), and involves characterizing an individual's tendency to negatively ruminate about their pain experiences (21, 48, 49). Unlike IAP which only quantifies whether or not attention is towards pain, PR has a negative affect (50, 51) component towards pain that quantifies negative thinking about pain experiences. The questions presented in the PCS-R have more emotional valence than the questions probed in the IAP protocol. This may explain why these two metrics were only marginally significantly positively correlated in the AS group and not significantly correlated in the HCs. As well, these findings are inconsistent with previous work in our lab that has shown that IAP scores and PCS scores in healthy individuals exhibit a modest positive trend (15). However, it is important to note that this study looked at scores generated from the entire PCS and not the relationship between IAP scores and PCS-R scores alone. The findings in the current study encourages the need for future work to investigate additional metrics that quantify attention to pain similarly to IAP but do not require an experimental pain stimulus and that do not capture pain affect as prominently as the PCS-R.

We also note that the experiences people are reflecting on when they complete the PCS-R or how long ago they occurred are not known. Someone with chronic pain who experiences persistent chronic pain could more readily recall their pain-related cognitions when completing the PCS-R. This assessment of their tendency to catastrophize (52) and/or ruminate about pain, could then better capture in the PCS-R a more accurate representation of their tendency to attend to pain. In contrast, because healthy individuals are not experiencing pain at the time of assessment, they may vary in how difficult it is to recall a pain experience and their pain-related cognitions when completing the PCS-R (52). This may impact how the PCS-R can capture their tendency to ruminate and attend to pain. This issue further highlights the importance to consider other metrics in future studies that can capture attention to pain and be used in both healthy individuals and people with chronic pain.

We did not find a relationship between the measures of attention to pain (IAP and PCS-R scores) and the performance measures of the NI task (ΔRTmean and ΔRTv). This was an unexpected finding considering our previous work has shown a significant positive correlation between IAP scores and ΔRTmean in healthy individuals (15). Both the A-P and IAP protocols are meant to capture an understanding of pain and attention interactions in individuals, but the lack of correlation suggests these two behavioural phenotypes reflect pain and attention interactions in different ways. IAP reflects a trait-like measure of attention to pain (15, 53). It is currently unknown whether the A and P type characterizations are trait or state-like designations of pain and attention interactions. However, consistent evidence of structural and functional brain region differences between A- and P-types (12–15) suggest that these behavioural characterizations are trait-like in nature. It is clear that more work needs to be done to investigate the trait or state like qualities of the A-P and IAP behavioral phenotypes in both healthy and chronic pain populations.

We also note a study limitation that should be addressed arising from examining AS is that it is a condition that is predominately found in males (54–56), and this has limited our ability to include an equal number of female participants and fully address any sex differences. There is a considerable amount of studies that suggest that chronic pain experiences and prevalence are different between males and females (57–63), and so it is important to observe whether there are differences in the reflection of pain and attention interactions between the sexes. Finally, a limitation is that our study did not include a non-painful stimulation control condition. Thus, we cannot factor out the possibility of a non-specific stimulation distraction effect impacting the participants' performance on our behavioural tasks. Our future iterations of these behavioural tasks will include control conditions that take this possibility into consideration. As well, our future studies will use larger sample sizes to explicitly examine sex differences and behavioural phenotypes in greater detail.

In conclusion, the current A-P/IAP behavioural phenotype characterization protocols are likely not appropriate for people with chronic pain as they do not account for the occurrence of chronic pain throughout their behavioural tasks. Although, PR could be used as a supplement to quantify attention to pain alongside IAP, other metrics are needed to be investigated that are more closely related to IAP and circumvent the use of experimental pain stimuli so that IAP phenotypes can be characterized in people with chronic pain. Attention-based therapies of chronic pain such as cognitive behavioural therapy (CBT) provide inconsistent success to help improve chronic pain (16, 17, 64–68), but a greater understanding of behavioural phenotypes of pain and attention interactions may contribute to a better identification of individuals most likely to benefit from attention-based chronic pain therapies such as CBT.

## Data Availability

The original contributions presented in the study are included in the article/**Supplementary Materials**, further inquiries can be directed to the corresponding author/s.
