# Supplementary material for: Can we characterize A-P/IAP behavioural phenotypes in people with chronic pain?
Source: Front Pain Res (Lausanne). 2023 Feb 15;4:1057659. doi: 10.3389/fpain.2023.1057659 (PMC9975728; doi:10.3389/fpain.2023.1057659)
Supplement: Supplementary file 1 [file Datasheet1.pdf]

Can we characterize A-P/IAP behavioural phenotypes in people with chronic pain?

Sanmugananthan VV, Cheng JC, Hemington KS, Rogachov A, Osborne NR, Bosma RL, Kim JA, Inman RD, and Davis KD

Supplementary Material

Supplementary Methods:

Painful stimulation was delivered at pre-determined intervals during the behavioural tasks. The stimulus application was automated using an Arduino microcontroller (Smart Project, Italy) which was connected to E-prime (where the behavioural tasks were coded) and the TENS device.[1]

Pain intensity ratings of the experimental stimulus intensity were checked and re-calibrated between runs of the behavioural experimental pain tasks if needed.

Transcutaneous Electrical Stimulation (TENS) Device Calibration

For each participant we established the electrical stimulus voltage (mV) that elicits a pain intensity score of approximately 50/100 (0-no pain, 100- worst pain imaginable) to be used in the behavioural tasks. Electrodes from the transcutaneous electrical nerve stimulation (TENS) device were placed on the participants' left volar forearm to stimulate the median nerve at 50Hz.[1] Stimulus intensity was slowly increased in increments of 0.5 millivolts (mV) until the participant verbally reported a pain intensity level between 40-60/100. This was repeated 3 times with a 1-minute interval between repetitions. We also checked that the stimulus intensity would provide the desired pain intensity over a sustained period similar to what would be used in the behavioural test. Thus, participants used a trackball to provide continuous ratings of pain intensity evoked by 20s of TENS at the voltage level established during the calibration procedure. This was repeated twice, with 30 seconds intervals between each repetition.[2]

Supplementary Analyses:

Pain Scores of the Ankylosing Spondylitis (AS) Group

The AS participants completed the painDETECT questionnaire, which is a screening-tool that can be used to detect the likelihood of neuropathic pain.[3,4] This questionnaire includes questions that assess current chronic pain at the time of testing and average pain over the past 4 weeks on a scale from 0 to 10 (0 =no pain, 10 = max pain). The group mean pain (+/- SD) at the time of testing was 2.2 +/- 2.0 and the average pain score over 4 weeks was 3.3 +/- 2.3. There were no significant differences in current pain scores at the time of testing between A-types and P-types (Mann-Whitney U test: A-types: M=2.0, SD=2.0, P-types: M=2.6, SD=2.0) (p=0.24, Cohen's d=0.32). There was only a marginally significant difference in average pain scores over 4 weeks between A-types and P-types,

such that P-types had overall higher pain-scores than the A-types (A-types: M=3.0, SD=2.5, P-types: M=4.2, SD=1.6) (p=0.056, Cohen's d=0.53).

Sex Difference Analyses

We examined the sex differences in the reaction time (RT) means and variances (RTv), and the intrinsic attention to pain (IAP) scores using independent-sample t-tests for the HCs and the AS group. We also examined the difference between PCS-R scores using a Mann-Whitney-U test between males and females for the HCs and the AS group.

Reactions Times

There were no sex significant differences in the mean RTs in the no-pain condition in HCs (t=0.95, p=0.35, Cohen's d=0.31) (males: M=1304.94 ms, SD=148.96 ms; females: M=1356.30 ms, SD=194.47 ms) or in the AS group (t=1.38, p=0.17, Cohen's d=0.46) (males: M=1364.76 ms, SD=169.03 ms; females: M=1449.57 ms, SD=217.81 ms) (Supplementary Figure 1). Also, there were no significant sex differences in RT in the pain condition for the HCs (t=1.39, p=0.17, Cohen's d=0.46) (males: M=1253.38 ms, SD=157.10 ms; females: M=1329.89 ms, SD=184.41 ms) or for the AS group (t=1. 64, p=0.11, Cohen's d=0.54) (males: M=1315.94 ms, SD=155.01 ms; females: M=1408.53 ms, SD=203.39 ms) (Supplementary Figure 2). Furthermore, there was no significant sex difference in RTv in the no-pain condition in HCs (t=0.63, p=0.53, Cohen's d=0.21) (males: M=9.3x10^4 ms^2, SD=3.4x10^4 ms^2; females: M=8.6x10^4 ms^2, SD=3.9x10^4 ms^2), or in the AS group (t=1.73, p=0.092, Cohen's d=0.57) (males: M=1.0x10^5 ms^2, SD=4.2x10^4 ms^2; females: M=1.3x10^5 ms^2, SD=5.7x10^4 ms^2) (Supplementary Figure 3), or for the HCs in the pain condition (t=0.18, p=0.86, Cohen's d=0.058) (males: M=8.6x10^4 ms^2, SD=3.0x10^4 ms^2; females: M= 8.4x10^4 ms^2, SD=3.0x10^4 ms^2) (Supplementary Figure 4). However, as shown in Supplementary Figure 4, there was a significant sex difference in task RTv in the AS group in the pain condition (t=2.06, p=0.046, Cohen's d=0.69) in that the females exhibited an overall higher mean RTv than the males (males: M=8.7x10^4 ms^2, SD=3.8x10^4 ms^2; females: M=1.1x10^5 ms^2, SD=4.4x10^4 ms^2).

Intrinsic Attention to Pain and Rumination Scores

There were no significant sex differences in IAP scores in HCs (t=0.68, p=0.50, Cohen's d=0.22) (males: M=-0.019, SD=0.78; females: M=0.15, SD=0.75) or in the AS group (t=2.0, p=0.053, Cohen's d=0.66) (males: M= -0.27, SD=0.85; females: M= 0.27, SD=0.75) (Supplementary Figure 5). Finally, there were no significant differences in the rumination subscale of the pain catastrophizing scale (PCS-R) scores in HCs (p=0.23, Cohen's d=0.47) (males: M=4.40, SD=3.39; females: M= 6.15, SD=4.38) or in the AS group (p=0.96, Cohen's d=0.11) (males: M=4.33, SD=4.01; females: M= 4.77, SD=4.25) (Supplementary Figure 6).

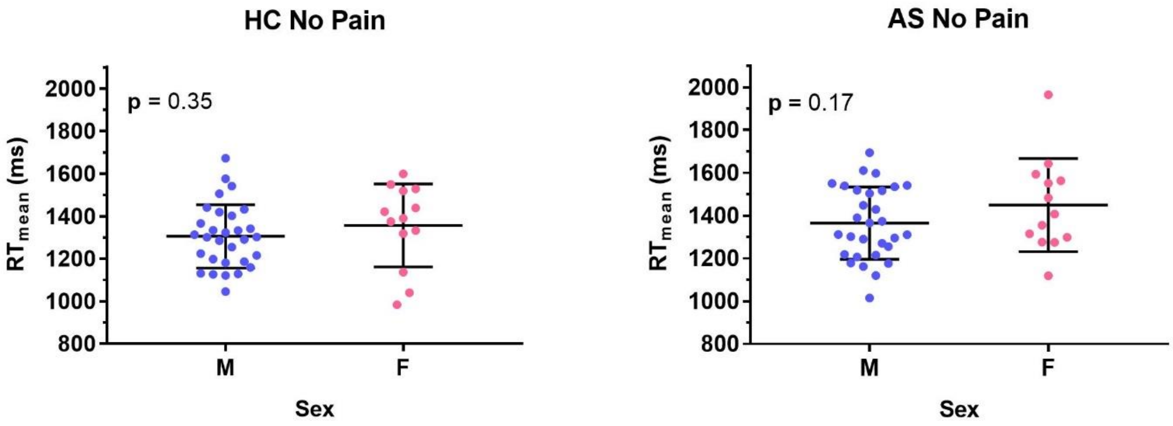

Supplementary Figure 1. Individual and mean group task reaction time (RT) across sexes in the healthy controls (HC) and ankylosing spondylitis (AS) group in the no-pain condition. The males are represented by the light blue dots. The females are represented by pink dots.

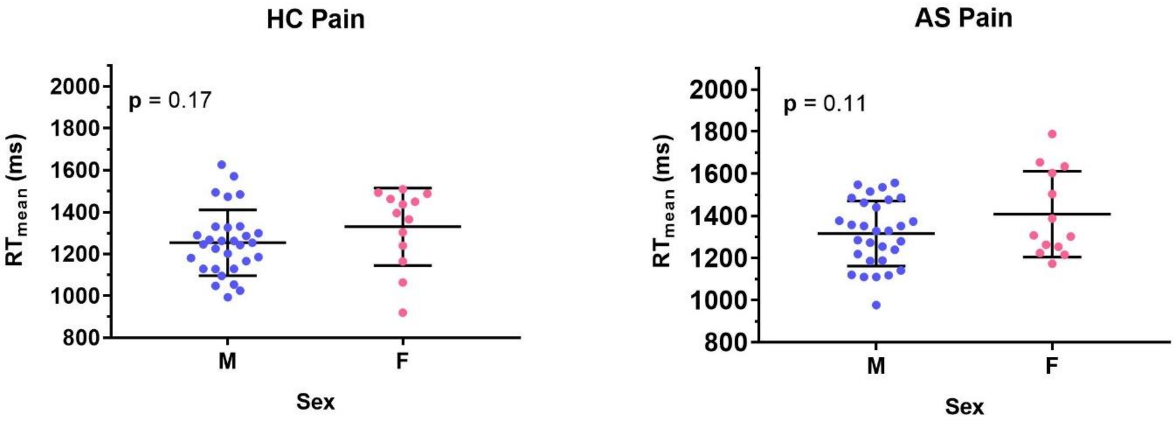

Supplementary Figure 2. Individual and mean group task reaction time (RT) across sexes in the healthy controls (HC) and ankylosing spondylitis (AS) group in the pain condition. The males are represented by the light blue dots. The females are represented by pink dots.

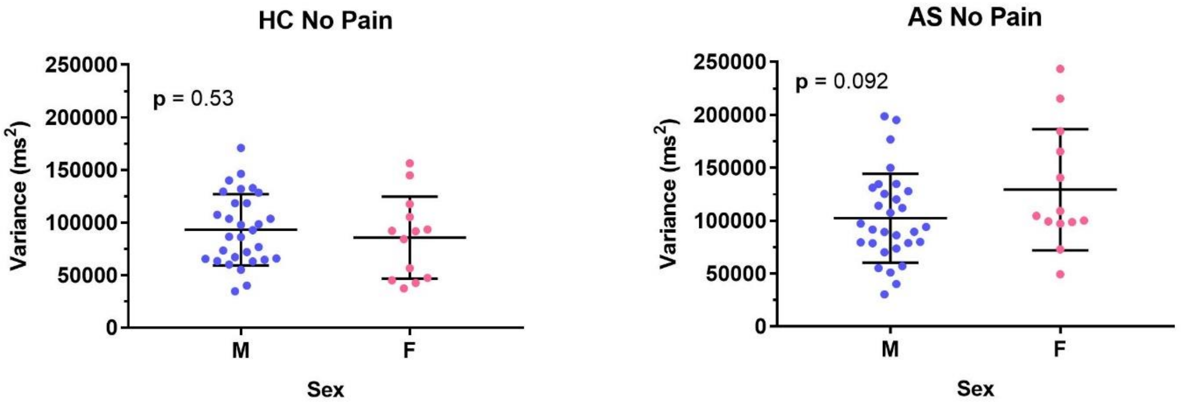

Supplementary Figure 3. Individual and mean group task reaction time variances (RTv) across sexes in the healthy controls (HC) and ankylosing spondylitis (AS) group in the no-pain condition. The males are represented by the light blue dots. The females are represented by pink dots.

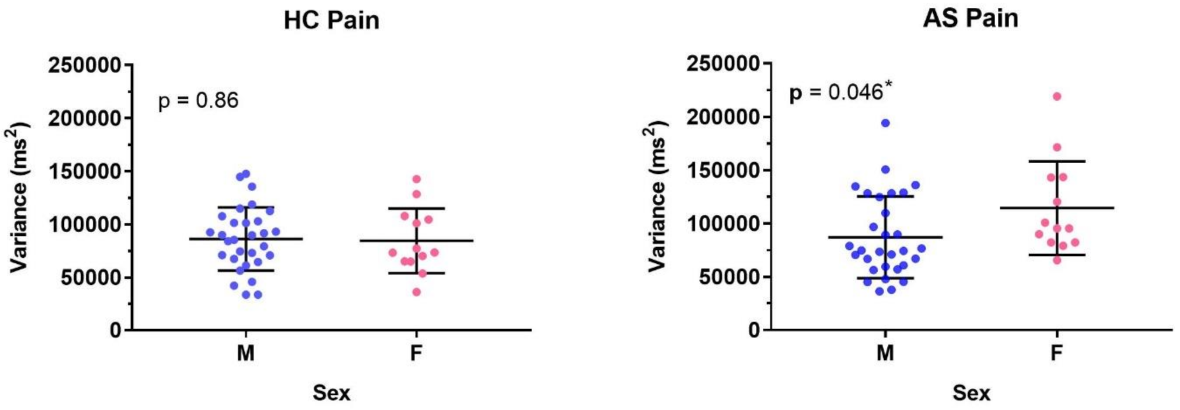

Supplementary Figure 4. Individual and mean group task reaction time variances (RTv) across sexes in the healthy controls (HC) and ankylosing spondylitis (AS) group in the pain condition. Higher task RTv was found in the females compared to the males in the AS group (right panel). The males are represented by the light blue dots. The females are represented by pink dots.

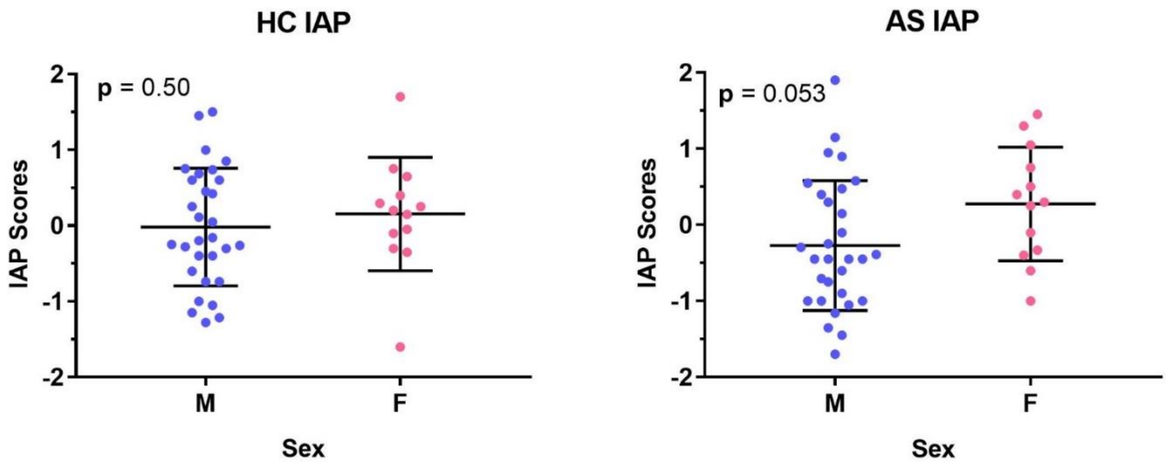

Supplementary Figure 5. Individual and group mean intrinsic attention to pain (IAP) across sexes in the healthy controls (HC) and ankylosing spondylitis (AS) group. The IAP scores were not significantly different between sexes in the HCs or AS group. The males are represented by the light blue dots. The females are represented by pink dots.

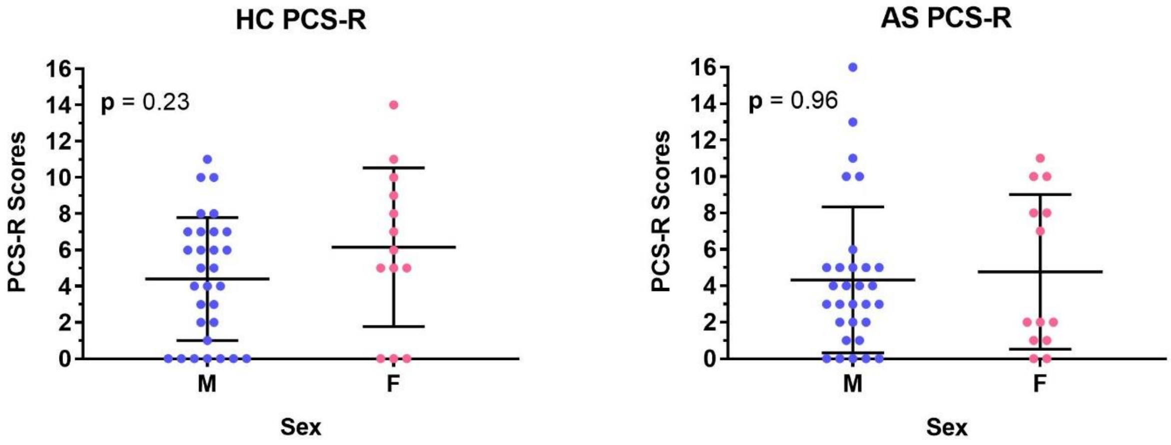

Supplementary Figure 6. Individual and group mean rumination scores across sexes in the healthy controls (HC) and ankylosing spondylitis (AS) group. The pain rumination scores from the rumination subscale of the pain catastrophizing scale (PCS-R) were not significantly different between sexes in the HCs or AS group. The males are represented by the light blue dots. The females are represented by pink dots

References:

1. Kucyi A, Salomons TV, Davis KD. Mind wandering away from pain dynamically engages antinociceptive and default mode brain networks. Proc Natl Acad Sci U S A. (2013) 110(46):18692–7. doi: 10.1073/pnas.1312902110

2. Cheng JC, Bosma RL, Hemington KS, Kucyi A, Lindquist MA, Davis KD. Slow-5 dynamic functional connectivity reflects the capacity to sustain cognitive performance during pain. NeuroImage. (2017) 157:61–8. doi: 10.1016/j.neuroimage.2017.06.005

3. Freynhagen R, Baron R, Gockel U, Tölle TR. painDETECT: a new screening questionnaire to identify neuropathic components in patients with back pain. Curr Med Res Opin. (2006) 22(10):1911–20. doi: 10.1185/030079906X132488

4. Wu Q, Inman RD, Davis KD. Neuropathic pain in ankylosing spondylitis: a psychophysics and brain imaging study. Arthritis Rheum. (2013) 65(6):1494–503. doi: 10.1002/art.37920
